# Single-cell sequencing of a novel model of neonatal bile duct ligation in mice identifies macrophage heterogeneity in obstructive cholestasis

**DOI:** 10.1038/s41598-023-41207-0

**Published:** 2023-08-29

**Authors:** Swati Antala, Kyle D. Gromer, Gaurav Gadhvi, Alyssa Kriegermeier, Jiao-Jing Wang, Hiam Abdala-Valencia, Joshua B. Wechsler, Harris Perlman, Deborah R. Winter, Zheng J. Zhang, Richard M. Green, Sarah A. Taylor

**Affiliations:** 1 Division of Pediatric Gastroenterology, Hepatology, and Nutrition, Department of Pediatrics, Ann and Robert H Lurie Children’s Hospital of Chicago, Chicago, IL USA; 2 Division of Rheumatology, Department of Medicine, Northwestern University, Chicago, IL USA; 3 Department of Surgery, Comprehensive Transplant Center, Northwestern University, Chicago, IL USA; 4 Division of Pulmonary and Critical Care Medicine, Northwestern University, Chicago, IL USA; 5 Division of Gastroenterology and Hepatology, Department of Medicine, Northwestern University, Chicago, IL USA; 6 Division of Gastroenterology and Hepatology, Department of Pediatrics, Children’s Hospital Colorado, University of Colorado School of Medicine, 13123 E. 16th Ave., Box B290, Aurora, CO 80045 USA; 7 Division of Hepatology, Department of Pediatrics, Kravis Children’s Hospital at Mount Sinai, New York, NY USA

**Keywords:** Innate immunity, Hepatology

## Abstract

Macrophages (MΦ) play a role in neonatal etiologies of obstructive cholestasis, however, the role for precise MΦ subsets remains poorly defined. We developed a neonatal murine model of bile duct ligation (BDL) to characterize etiology-specific differences in neonatal cholestatic MΦ polarization. Neonatal BDL surgery was performed on female BALB/c mice at 10 days of life (DOL) with sham laparotomy as controls. Comparison was made to the Rhesus Rotavirus (RRV)-induced murine model of biliary atresia (BA). Evaluation of changes at day 7 after surgery (BDL and sham groups) and murine BA (DOL14) included laboratory data, histology (H&E, anti-CD45 and anti-CK19 staining), flow cytometry of MΦ subsets by MHCII and Ly6c expression, and single cell RNA-sequencing (scRNA-seq) analysis. Neonatal BDL achieved a 90% survival rate; mice had elevated bile acids, bilirubin, and alanine aminotransferase (ALT) versus controls (p < 0.05 for all). Histology demonstrated hepatocellular injury, CD45+ portal infiltrate, and CK19+ bile duct proliferation in neonatal BDL. Comparison to murine BA showed increased ALT in neonatal BDL despite no difference in histology Ishak score. Neonatal BDL had significantly lower MHCII-Ly6c+ MΦ versus murine BA, however, scRNA-seq identified greater etiology-specific MΦ heterogeneity with increased endocytosis in neonatal BDL MΦ versus cellular killing in murine BA MΦ. We generated an innovative murine model of neonatal obstructive cholestasis with low mortality. This model enabled comparison to murine BA to define etiology-specific cholestatic MΦ function. Further comparisons to human data may enable development of immune modulatory therapies to improve patient outcomes.

## Introduction

Pediatric cholestatic liver disease is a significant cause of morbidity and mortality and remains the leading indication for pediatric liver transplantation^1^. Cholestatic disorders accounted for 43% of all liver transplants in children less than 18 years of age in 2021 of which biliary atresia (BA) comprised 93% (based on Organ Procurement and Transplantation Network data as of 4/1/2022). BA is a form of obstructive cholestasis for which the mechanism includes initial damage to large bile ducts followed by injury to smaller upstream bile ducts induced by bile salt toxicity and inflammation^2^. However, large therapeutic gaps exist, as there are no established medical therapies to effectively slow disease progression, particularly in children^1^.

Macrophages are a critical immune cell in the pathogenesis of cholestatic liver disease, however, the role for specific subsets has not been well characterized. Macrophages are a heterogeneous and plastic cell population that respond to specific environmental cues, e.g. bile acids, to modulate the immune response^3,4^. In the initiation of liver injury, disease associated molecular patterns (DAMPs) can stimulate tissue resident macrophages, i.e. Kupffer cells, or induce recruitment of bone marrow monocyte-derived macrophages to the liver. As liver injury progresses, macrophages adopt different functional states involved in inflammation, fibrosis, and tissue repair demonstrating the importance of macrophage phenotypic and functional heterogeneity in liver disease progression and resolution^5^.

Previous studies using the murine model of bile duct ligation (BDL) have demonstrated a central role for macrophages in the immune response to obstructive cholestasis and subsequent liver injury^6^. More specifically, studies in murine BDL have shown that macrophages contribute to both the progression and resolution of fibrosis in the setting of obstructive cholestasis ^7^. In line with these studies, we previously established an innovative model of reversible BDL and demonstrated elevated hepatic *CD68* in mice both after BDL alone, and after BDL with reversal and improved liver injury^8^. This prior work demonstrates the importance of determining both the subset-specific and stage-specific macrophage functions in cholestasis. However, it is unknown how macrophages differ based on the etiology of cholestasis. Multiple studies demonstrate a role for macrophages in the well-established rhesus rotavirus (RRV)-induced murine model of biliary atresia^9–12^, however, a non-immune neonatal murine model of biliary obstruction is lacking. This gap in knowledge prevents mechanistic studies necessary to identify disease-specific immune modulatory therapeutic targets.

In the present study we aim to establish a novel murine model of neonatal BDL and compare this model to the well-established RRV-induced model of murine BA. We identify etiology-specific differences in the macrophage immune response to biliary obstruction including distinct transcriptional phenotypes. Ongoing mechanistic studies with this novel neonatal model of BDL will improve our understanding of the heterogeneous immune response between etiologies of obstructive pediatric cholestatic liver disease to ultimately improve medical therapies.

## Materials and methods

### Characterization of a novel murine model of neonatal BDL

**Figure 1:**
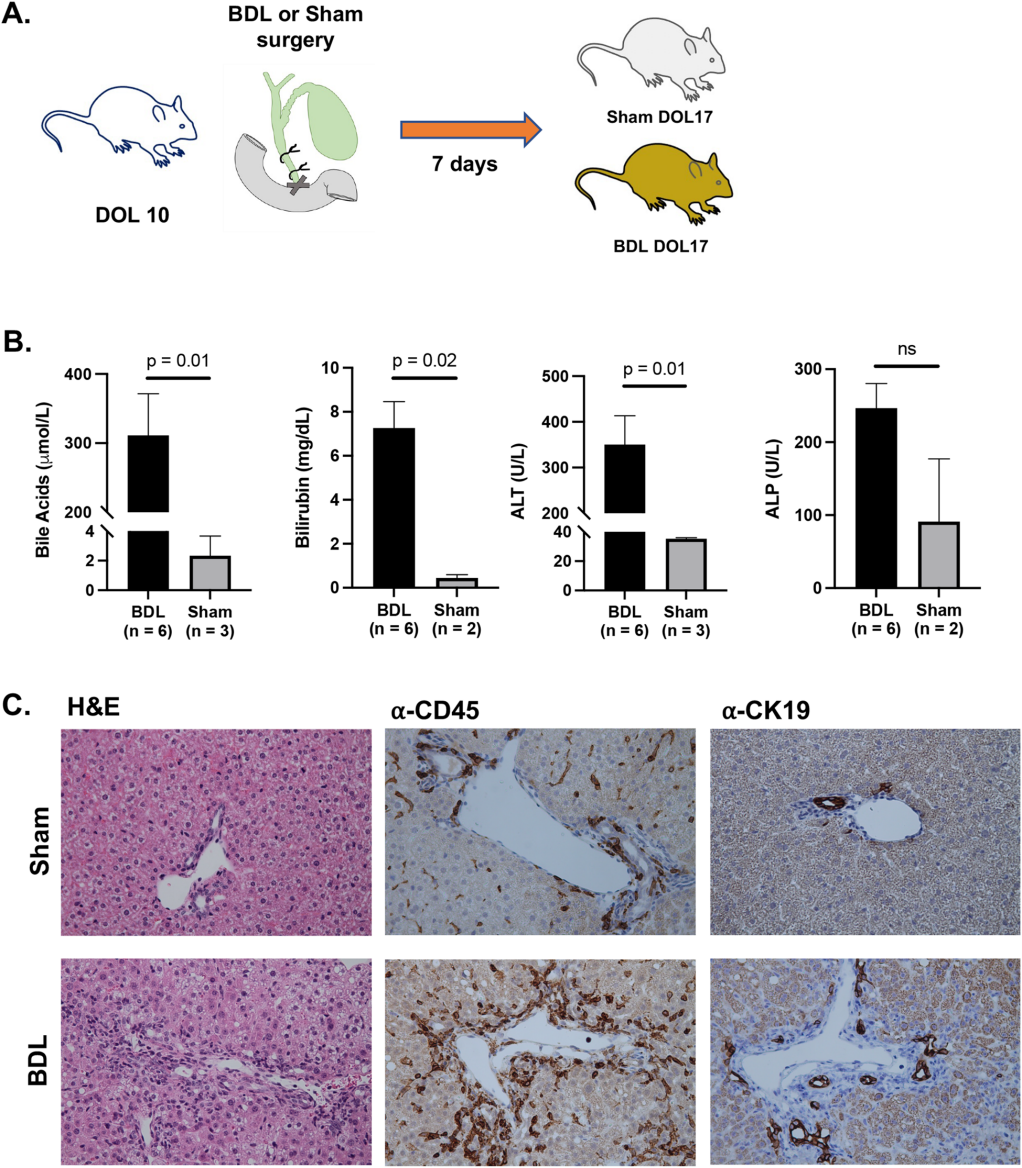
**(A)** Overview of neonatal BDL model with sham controls. (**B**) Comparison of biochemical markers between neonatal BDL mice and controls shows evidence of liver injury and cholestasis in neonatal BDL. (**C**) Representative H&E images and staining with anti-CD45 and anti-CK19 at 20x magnification shows hepatocellular injury, CD45+ portal infiltrate, and proliferation of CK19+ bile ducts.

This study was carried out following the recommendations in the Guide for the Care and Use of Laboratory Animals of the National Institutes of Health. All procedures and protocols were approved by the Northwestern University Institutional Animal Care and Use Committee Office and the study is reported in accordance with ARRIVE guidelines. Surgical ligation of the common bile duct was performed on individual female BALB/c mice at day of life (DOL) 10 following previously established technique within the Northwestern Microsurgery and Preclinical Research Core Facility (Fig. 1A)^8, 13^. Briefly, a midline incision was made to enter the peritoneal cavity and the common bile duct was exposed and separated from the adjacent portal vein and hepatic artery. An 8-0 nylon suture was used to tie two surgical knots and ligate the bile duct. The abdomen was then closed in two layers. Sham surgery was performed on control mice at DOL 10 by creating an abdominal incision and closure without further manipulation. All surgery was done using isoflurane inhalation for anesthesia, and efforts were made to minimize suffering.

Blood and liver tissue were obtained 7 days after surgery (DOL 17) from both BDL and sham mice. Levels of bile acids (BA), alanine aminotransferase (ALT), alkaline phosphatase (ALP), and total bilirubin (TB) in blood were measured in both experimental groups. Paraffin-embedded liver tissue was stained by the Northwestern University Mouse Histology and Phenotyping laboratory using Hematoxylin and Eosin (H&E), anti-CD45 antibody (Abcam, ab10558), and anti-cytokeratin 19 antibody (CK19; Developmental Studies Hybridoma Bank, TROMA-III).

### Comparison of hepatic immune injury in neonatal BDL versus murine BA

We next compared the level of liver injury in our neonatal murine model of BDL to the well-established RRV-induced murine model of BA and saline-injected controls^14, 15^. In the murine model of BA, newborn BALB/c mice received intra-peritoneal injections of RRV at DOL 0 and developed sequelae of biliary obstruction by DOL 10–14 with similar mortality rates up to 20–30% as previously reported^14–17^. Blood and liver tissue were collected from murine BA and control mice at DOL 14; laboratory and histology analyses were performed as described above for neonatal BDL. Due to known variability in disease penetrance in the RRV-induced murine model of BA, laboratory values were included for RRV mice that achieved bile acids > 10 µmol/L.

To better evaluate differences in parenchymal liver injury between experimental models, Ishak scoring was performed on H&E slides from five neonatal BDL mice and four RRV mice by two blinded investigators (RG and AK) as previously described for the adult model of murine BDL^8^. Scores were assigned from 0 to 4 for periportal or periseptal interface hepatitis, 0–6 for confluent necrosis, 0–4 for focal lytic necrosis, and 0–4 for portal inflammation^18^. The average score per category for the two reviewers was obtained, the cumulative Ishak grade (range of 0 to 18) was calculated, and then scores were compared between experimental groups.

Next we performed multiparameter flow cytometry to define the hepatic macrophage cell subsets involved in liver injury for each experimental group (n = 4 for RRV and saline groups, n = 5 for BDL and sham groups)^19^. To establish a single cell suspension from whole mouse liver, we cardioperfused each mouse using phosphate-buffered saline and removed the liver. The liver was cut into small pieces and placed into a gentleMACS C Tube (Miltenyi Biotec) with 2.5 mL digestion buffer [DNase I (Sigma), Liberase TL (Sigma), and HBSS (Gibco)]. Mechanical digestion was accomplished using both the Miltenyi Biotec gentleMACS Dissociator and incubation with shaking at 37 °C for 30 min. The digested tissue was then strained through a 40 μm filter at 4 °C into a 50 mL conical tube and Pharm Lyse was used to lyse remaining red blood cells. Samples were spun at 350 rcf for 7 min (4 °C) and cell counts were obtained. CD45 enrichment was then performed following the established protocol for CD45 MicroBeads and magnetic separation using the MACS Separator and MS columns from Miltenyi Biotec. We next stained our single cell suspension with a panel of 12 antibodies for the following cell surface markers: CD45, Siglec-F, Ly6g, CD3, CD19, CD11b, CD64, Ly6c, and CD11c (Supplemental Table 1). The BD FACSAria at the Robert H. Lurie Comprehensive Cancer Center Flow Cytometry Core Facility was utilized for flow cytometry. We established the gating strategy shown in Supplemental Fig. 1 through use of fluorescence minus one (FMO) controls. We identified CD11b+/CD64+ macrophages and compared subsets by level of expression for MHCII and Ly6c. We labeled cells with high cell surface expression of MHCII and Ly6c as MHCII+ and Ly6c+ respectively.

### scRNA-seq analysis of murine macrophages in neonatal obstructive cholestasis

We first isolated CD45+/CD3−/CD19−/Siglec-F−/Ly6g− immune cells from three pooled livers per experimental group of female BALB/c mice: DOL 17 neonatal BDL, DOL 14 murine BA, and non-diseased DOL14 healthy controls without saline or RRV injection. scRNA-seq libraries were prepared using the Single Cell 3’ v3 Reagent Kit and the 10× Genomics Chromium Controller in the Northwestern Next Generation Sequencing Facility, and sequencing was performed on the Illumina HiSeq 4000 (healthy control and murine BA) and NovaSeq 6000 (neonatal BDL) platforms. Raw sequence data was processed using the 10× Genomics Cell Ranger 3.1.0 (healthy control and murine BA) and 6.0.0 (neonatal BDL) pipelines to identify 8172 cells in healthy controls, 5509 cells in neonatal BDL, and 5185 cells in murine BA (Supplemental Fig. 2A).

To compare differences in macrophage heterogeneity between murine models, we analyzed single cell libraries using the Seurat v 4.1.0 R toolkit following a similar workflow to our previously published data on human liver scRNA-seq data^20–22^. Briefly, we filtered cells with gene counts between 200 and 5000, and < 20% mitochondrial gene content (Supplemental Fig. 2B). We normalized and scaled our data using the functions NormalizeData, FindVariableFeatures, and ScaleData. We plotted each dataset by principal component analysis (RunPCA function) and evaluated the variability in each principal component by the ElbowPlot function to determine the number of dimensions for clustering. Based on this workflow we clustered the cells using the FindNeighbors and FindClusters functions by the following parameters: ten dimensions with 0.2 resolution for healthy control, and ten dimensions with 0.3 resolution for both neonatal BDL and murine BA. Cell clusters were visualized by Uniform Manifold Approximation and Projection (UMAP) using the function RunUMAP and canonical cell markers were used to identify each immune cell subset (Supplemental Figs. 3, 4, 5). Our flow cytometry gating strategy to isolate CD45+ /SigF−/Ly6g−/CD3−/CD19− produced immune cells enriched for mononuclear phagocytes while excluding most T cells, B cells, eosinophils, and neutrophils. Canonical cell markers chosen for annotation thereby included: *Cd14*, *Fcgr3*, and *Cd63* (macrophages); *Itgax*, *Clec9a*, and *Siglech* (dendritic cells); *Ly6g*, *CD63*, and *Stfa2* (neutrophils); *Nkg7* (T cells); *Pax5* and *Mzb1* (B cells); *Cd36* (endothelial cells). We further confirmed our cell cluster assignments using SingleR and the Immgen database ^23, 24^.

Similar to our prior work on hepatic macrophages in children with cholestasis^21^, we integrated macrophages from both diseased murine models to identify common cholestatic macrophage subsets. This new dataset included macrophages from neonatal BDL (clusters 0, 1, 2, 3, and 5) and from murine BA (clusters 0, 1, 3, 4, and 5). Integration was accomplished using the functions SelectIntegrationFeatures, FindIntegrationAnchors, and IntegrateData. Clustering of the integrated dataset was then performed following the same steps as described above, and conserved genes within each integrated macrophage cluster were determined using the function FindConservedMarkers. We next characterized the enriched processes by macrophage cluster using Gene ontology (GO) enrichment analysis of the differentially expressed genes for each cluster with the remainder of genes within the cluster as background^25, 26^. Integrated murine cholestatic macrophages were also compared to non-diseased murine macrophage clusters, and differences were visualized by volcano plot and Pearson correlation of shared genes (Morpheus, https://software.broadinstitute.org/morpheus). Lastly, we compared the expression level of genes identified in our murine macrophages to their orthologous human genes in pediatric cholestatic liver macrophages^21^.

### Statistical analysis and data availability

Sample size determinations were made to provide a descriptive characterization of the neonatal BDL model. To minimize bias, mouse pups were randomly assigned an experimental group by the investigators and all litters were treated similarly to minimize confounders. Due to overt differences in experimental conditions, the investigators were not blinded. Differences in laboratory values, histology scores, and flow cytometry counts were determined using t-test and ANOVA with Bonferroni post-hoc test for pairwise and multi-group comparisons as appropriate. Means with standard error of the mean (SEM) are reported and significance was defined as p-value < 0.05. Outliers were removed from laboratory data by group using the ROUT method with q-value of 1% in GraphPad Prism. Data visualization was performed using GraphPad Prism 9 software. FlowJo 10.6.2 software was used for analysis of all flow data, and count beads (123count eBeads Counting Beads, Invitrogen, Catalog # 01-1234-42) were used to determine cell numbers. Averages of cell counts among replicates were taken and then compared between groups. The ratio of cell populations between each mouse model and its respective control was obtained for comparison between experimental models.

All scRNA-seq analysis was performed using RStudio version 1.2.1335. Differentially expressed genes used for comparison between clusters were defined as those with average log twofold change > 1 and p-value < 0.05. The degree of similarity between cholestatic and non-diseased clusters was assessed by correlation analysis of genes common between clusters (Morpheus, https://software.broadinstitute.org/morpheus). Processes identified by GO enrichment analysis were considered significant if they had an FDR-corrected p-value < 0.05, enrichment score > 3, and at least 10 genes identified in the intersection with the GO process. scRNA-seq data is available through the Gene Expression Omnibus data repository by accession GSE228996.

## Results

### BDL in a neonatal mouse is a novel model of non-immune biliary obstruction

BDL was performed on a total of 29 neonatal mice with an overall survival rate of 90% at 7 days after surgery. Blood chemistry demonstrated significantly increased mean bile acids (p = 0.01), and bilirubin (p = 0.02) in neonatal BDL compared to sham controls (Fig. 1B). Mean bile acids and bilirubin were 311 +/- 60.1 μmol/L and 7.27 +/- 1.20 mg/dL in neonatal BDL compared to 2.33 +/- 1.33 μmol/L and 0.45 +/- 0.15 mg/dL in sham respectively. Mean ALT was also significantly increased in neonatal BDL at 351 +/- 63.0 U/L compared to 35.5 +/- 0.67 U/L in sham controls (p = 0.01). Alkaline phosphatase did not significantly differ between groups with mean 247 +/- 33.6 U/L in BDL mice and 91.0 +/- 86.0 U/L in controls (p = 0.08).

In addition to laboratory findings, changes on hepatic histology also showed features of obstructive cholestasis in our neonatal model of BDL (Fig. 1C). Staining by H&E showed portal tract expansion with hepatocyte damage, inflammation, lobular disarray, and focal areas of parenchymal necrosis. Prominent immune cell infiltrate was further highlighted by staining with anti-CD45 antibody. Lastly, bile duct proliferation was observed as demonstrated by anti-CK19 staining.

### Biochemical and histological comparison between neonatal BDL and murine BA

**Figure 2:**
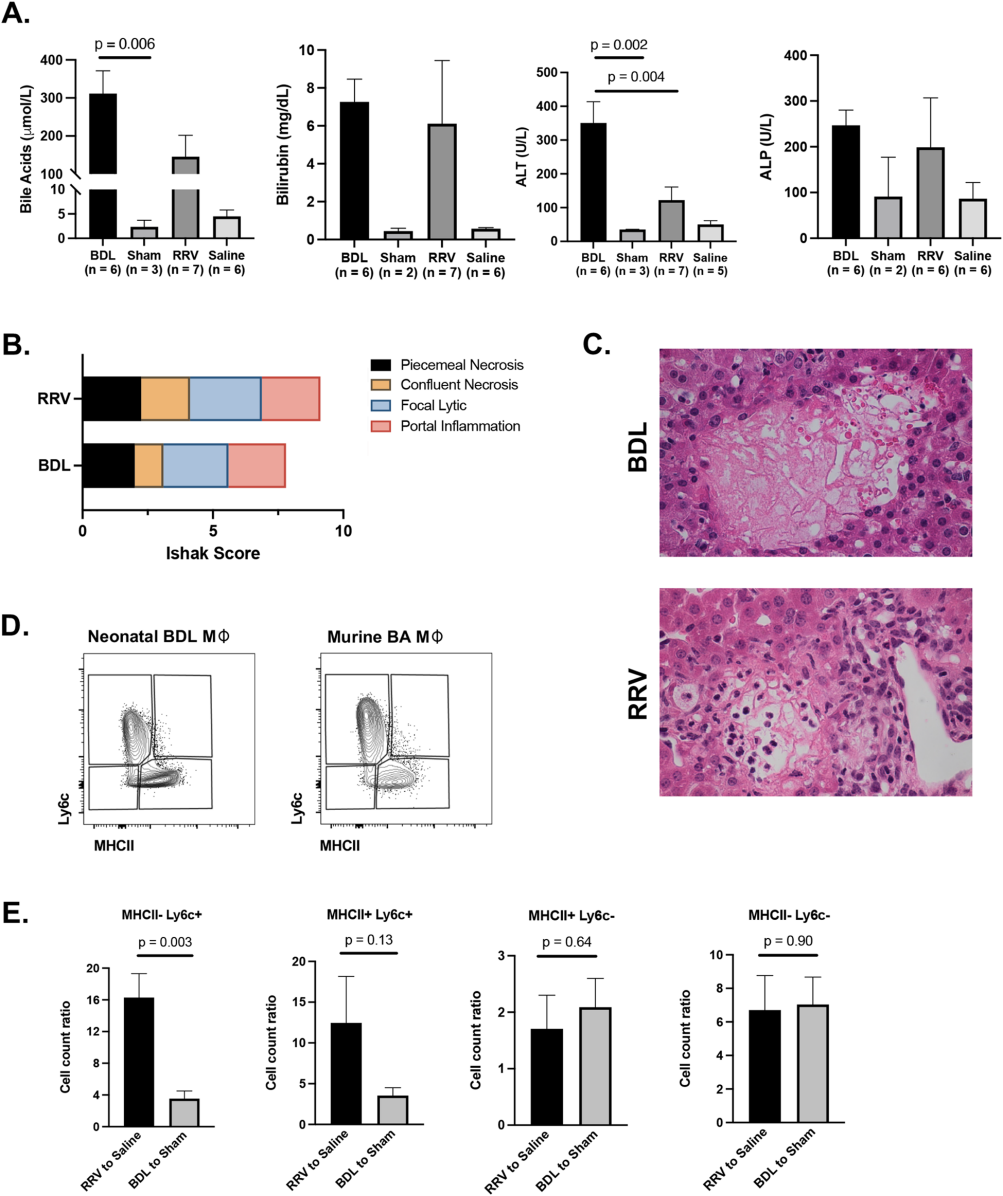
**(A)** Comparison of laboratory data across neonatal BDL and murine BA mice showed increased ALT in neonatal BDL. (**B, C**) Ishak scoring did not differ between the experimental models of obstructive cholestasis with similar areas of necrosis in each model. (**D, E**) Flow cytometry analysis of macrophage subsets showed only a significant difference in MHCII-Ly6c+ macrophages between models.

We next evaluated how the severity of liver injury may differ between neonatal BDL and murine BA using a combination of laboratory data and histology findings. Comparison across all experimental models and their respective controls showed that ALT was significantly higher in neonatal BDL with mean 351 +/- 63.0 U/L versus 122 +/- 38.7 in murine BA (p = 0.0004; Fig. 2A). In contrast, bilirubin, bile acids, and alkaline phosphatase did not differ between experimental groups (Fig. 2A).

To further determine if the type of parenchymal liver injury may differ between etiology of obstructive cholestasis, we compared Ishak scores between the 2 experimental groups. BDL mice had an overall mean Ishak grade of 7.8 +/- 0.87 compared to 8.9 +/- 0.24 in murine BA (p = 0.32). Neither total Ishak grade nor individual component scores significantly differed between groups (Fig. 2B, C). This finding supports the ability for our murine model of neonatal BDL to produce a similar level of hepatic injury as murine BA despite a different cause of biliary obstruction.

### Flow cytometry analysis identifies differences in macrophage subsets by disease model

As macrophages are known to play a critical in the hepatic immune response to obstructive cholestasis, we compared the number of CD64+ macrophage subsets by experimental model relative to their respective control (Fig. 2D, E). We identified a significant increase in the proportion of MHCII-Ly6c+ macrophages in murine BA when compared to BDL (p = 0.01). In contrast, MHCII-Ly6c−, MHCII+ Ly6c+, and MHCII+ Ly6c− macrophages did not significantly change in number between experimental models (p > 0.05 for each comparison). This data supports possible differences in either macrophage cell recruitment and/or hepatic polarization of macrophage subsets based on the etiology of obstructive cholestasis.

### scRNA-seq analysis identifies murine cholestatic macrophage subsets with distinct transcriptional signature from non-diseased neonatal BALB/c mice

We used scRNA-seq to better define the functional heterogeneity of macrophages across murine models versus controls and enable comparison to our previously published scRNA-seq data from human pediatric cholestatic liver^21^. Clustering of CD45+/SigF−/Ly6g−/CD3−/CD19− immune cells enriched for mononuclear phagocytes identified macrophages in each murine model. More specifically, this analysis resulted in annotation of three macrophage clusters in non-diseased neonatal BALB/c liver, and five macrophage clusters in both neonatal BDL and murine BA samples (Supplemental Figs. 3, 4, 5 and Supplemental Table 2). Top differentially expressed genes by macrophage cluster in non-diseased mice included: immune regulatory genes of *Vsig4*, *Cd5l*, and *Marco* in normal macrophage cluster 1; genes encoding heat shock proteins *Hspa1b* and *Hspa1a* in normal macrophage cluster 3; inflammatory genes *S100a6*, *S100a8*, and *Ccl4* in normal macrophage cluster 6 (Supplemental Fig. 3D).

To characterize common murine cholestatic macrophage functions, we performed integrated analysis of macrophages across disease models and first compared overall gene expression of murine cholestatic macrophages to non-diseased macrophages. Murine cholestatic macrophages demonstrated reduced expression for the tissue-resident gene *Clec4f* and transcriptional factors known to regulate macrophage polarization such as *Junb*, *Klf2*, and *Klf6* ( Supplemental Fig. 3E)^27–29^. In addition, diseased macrophages expressed more pro-inflammatory processes corresponding to genes such as *S100* genes, *Ccl5*, and *Ccl2* ( Supplemental Fig. 3E).

**Figure 3:**
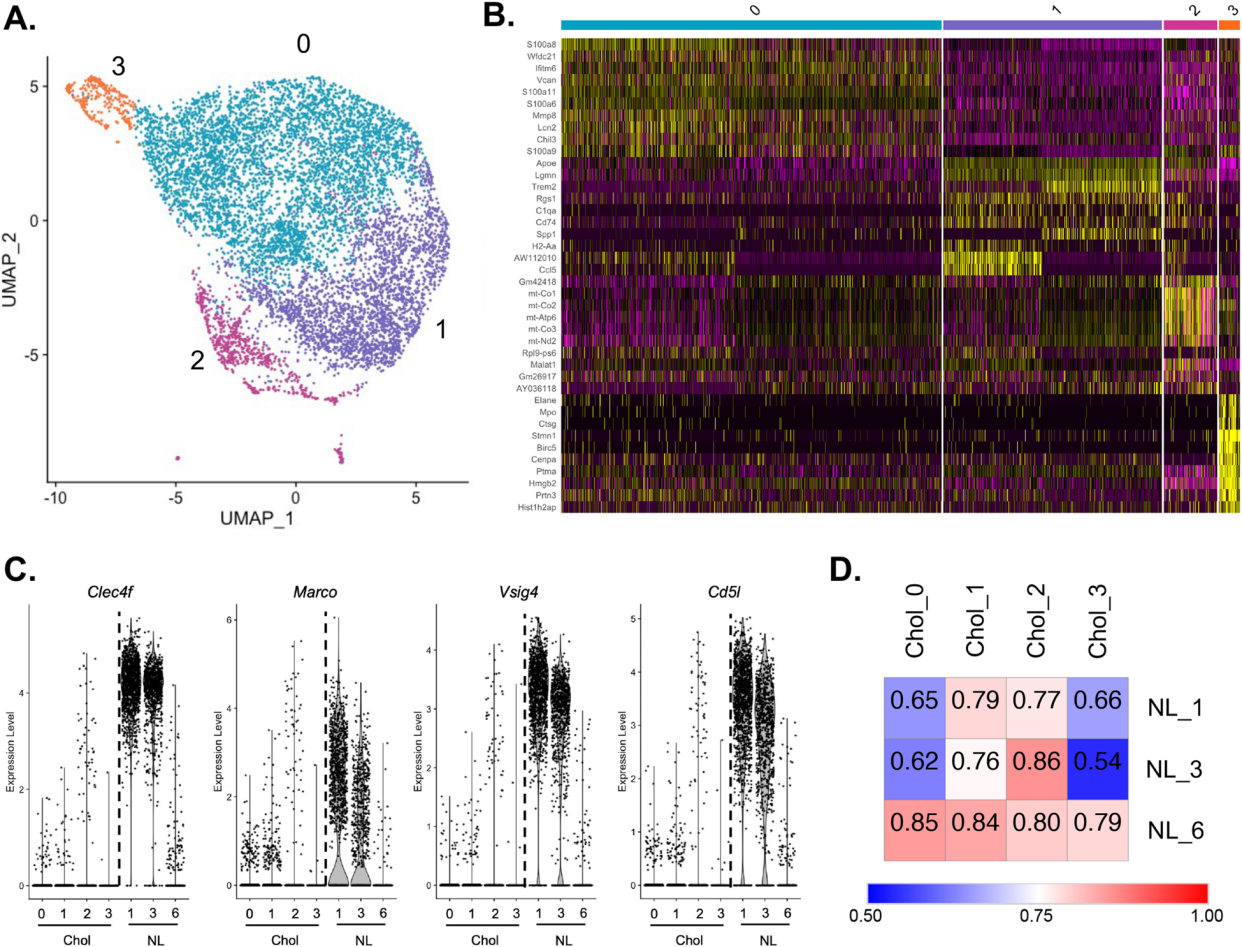
(**A**) UMAP of integrated murine cholestatic macrophages shows 4 distinct macrophage subsets. (**B**) Heatmap illustrates top 10 differentially expressed genes by cholestatic macrophage cluster. (**C**) Subset-specific comparison of cholestatic macrophages (Chol) to non-diseased macrophages (NL) demonstrated lower expression of *Clec4f *and immune regulatory genes (*Marco*, *Vsig4*, and *Cd5l*) across cholestatic macrophage subsets. (**D**) Pearson correlation showed that one non-diseased macrophage cluster (NL_6) had greater expression of inflammatory genes and was most similar to murine cholestatic macrophages.

Further clustering of the integrated cholestatic macrophages identified four distinct subsets (Fig. 3A, B Supplemental Table 3). Cluster-specific comparisons between the integrated cholestatic macrophage subsets and non-diseased macrophages showed one cluster (normal cluster 6) that expressed greater levels of *S100a6* and *S100a8,* similar to diseased macrophages (Supplemental Fig. 3D). Importantly, the other two non-diseased macrophage clusters demonstrated increased expression for immune regulatory genes (e.g. *Marco*, *Vsig4*, *Cd5l*) in comparison to normal cluster 6 and all cholestatic macrophage subsets (Fig. 3C). Pearson correlation further defined the similarity between the cholestatic macrophages and normal inflammatory macrophage cluster 6, thereby supporting the presence of pro-inflammatory macrophages in normal conditions similar to previous data in humans (Fig. 3D)^30^. Of the four cholestatic macrophage subsets, cluster 2 was most similar to a non-inflammatory normal macrophage cluster with Pearson correlation of 0.86 suggesting some cholestatic macrophages may be derived from their non-diseased counterparts (Fig. 3D).

### Murine cholestatic macrophages exhibit a similar transcriptional profile to human cholestatic macrophages

Cluster-specific transcriptional signatures of murine cholestatic macrophages are shown in Fig. 3B. This analysis demonstrated that murine cholestatic macrophage cluster 0 was enriched for *S100* genes and *Vcan* similar to our previously identified human cholestatic monocyte-like macrophages^21^. Additionally, murine cholestatic macrophage cluster 1 had increased expression for *Apoe*, *Lgmn*, and *Trem2,* genes previously identified in human lipid-associated macrophages^21, 31^. Comparison of murine macrophage clusters by etiology to previously identified human cholestatic macrophage genes from BA and non-BA patients is shown in Supplemental Fig. 6. No murine macrophage cluster expressed genes similar to previously identified adaptive macrophages^21^.

Cholestatic macrophage cluster 2 had increased expression for mitochondrial genes and the long noncoding RNA *Malat1* that has been associated with autophagy, suggesting a subset of macrophages under higher levels of cellular stress^32^. Lastly, cholestatic macrophage cluster 3 was enriched for genes associated with defense response and pathogen elimination such as *Ctsg* and *Mpo*^33, 34^. Taken together, these findings show subset-specific similarities in human and murine cholestatic macrophage polarization that may enable future translation studies.

### Murine cholestatic macrophages exhibit disease-specific function

**Figure 4:**
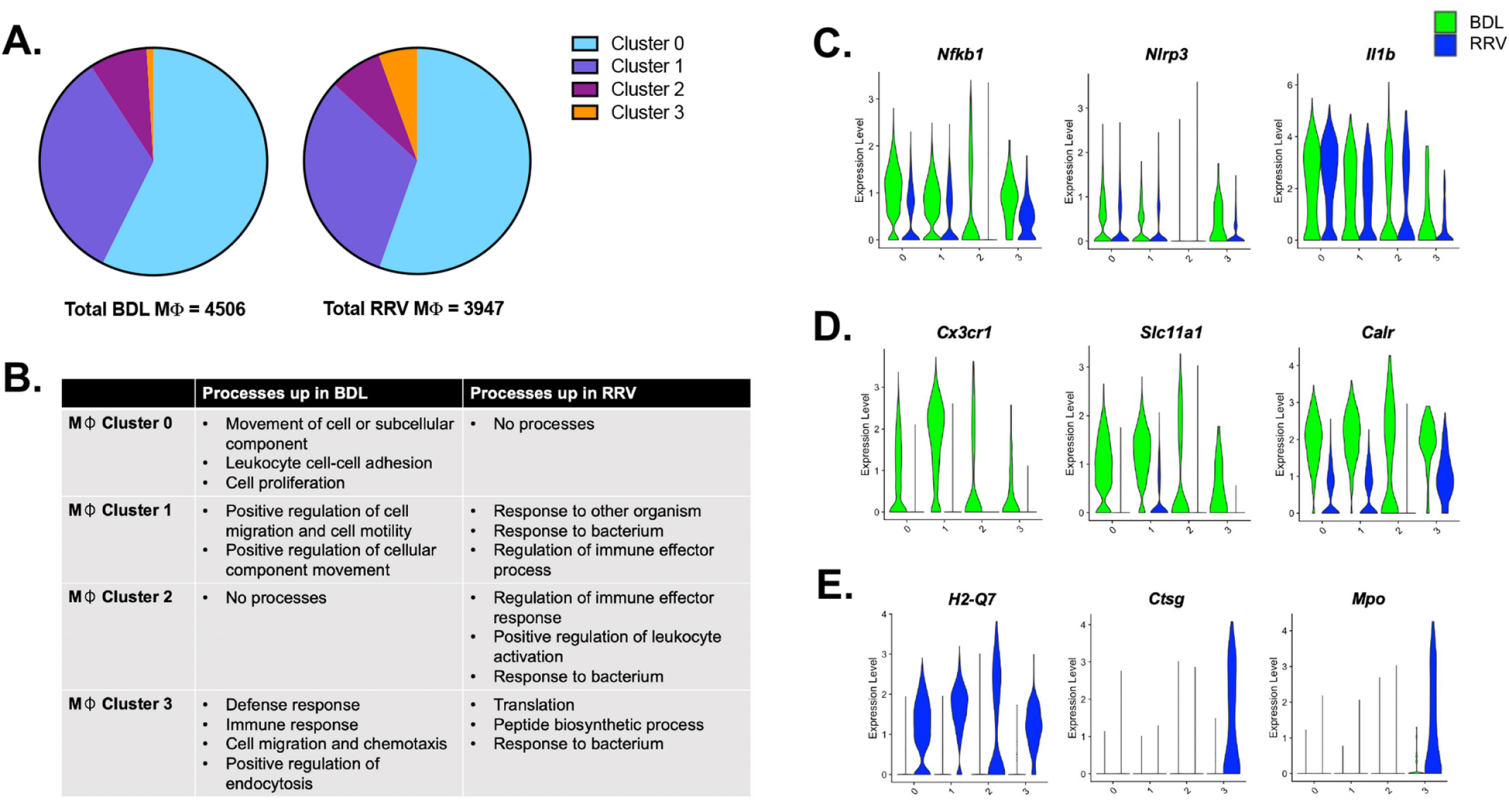
(**A**) The contribution of each cholestatic macrophage cluster to total macrophages for each model showed increased numbers of macrophage cluster 3 in murine BA. (**B**) Representative GO processes that were enriched for each macrophage cluster by experimental model are shown. (**C**) Both cholestatic murine models showed high levels of genes involved in NF-kB and inflammasome signaling, however, neonatal BDL mice showed increased expression for genes involved in endocytosis (**D**) whereas murine BA showed increased expression for genes involved in antigen presentation and pathogen degradation (**E**).

To determine how the four murine cholestatic macrophage subsets may differ by etiology of obstructive cholestasis, we compared the cluster-specific transcriptional signatures by disease model. Among the four clusters, cholestatic macrophage cluster 3 differed most in number and was increased in the RRV-induced murine model of BA (Fig. 4A). Representative differences in enriched GO processes are shown in Fig. 4B. Cholestatic macrophage cluster 3 showed enrichment for translation, biosynthetic processes, and response to bacterium in murine BA as compared to defense response, cell migration, and endocytosis in neonatal BDL (Fig. 4B). Cholestatic macrophage clusters 0 and 1 also showed enrichment for processes of cell movement and migration in neonatal BDL. In contrast, murine BA macrophages from clusters 1 and 2 demonstrated enrichment for regulation of immune effector processes in addition to response to bacterium.

Our findings by GO enrichment analysis are further supported by differences in specific genes shown in Fig. 4C–E. While both disease models had similar expression for genes involved in the NF-kB pathway and inflammasome signaling (*Nfkb1*, *Nlrp3*, *Il1b*)^35, 36^, alternate immune pathways differed by disease model. For example, macrophages in neonatal BDL showed greater expression for the chemokine *Cx3cr1* and genes associated with phagocytosis (*Slc11a1*, *Calr*)^37, 38^. In contrast, murine BA macrophages showed greater expression of specific genes involved in antigen presentation such as *H2-Q7* despite no differences in overall MHCII cell surface expression by our flow cytometry analysis. Furthermore, macrophage cluster 3 exhibited greater levels of *Ctsg* and *Mpo* in murine BA, genes involved in pathogen destruction^33, 34^. These data overall highlight the role for disease-specific macrophage heterogeneity in the immune mechanism of pediatric cholestasis that may impact the response to emerging medical therapies.

## Discussion

In the present study, we have successfully established a novel neonatal murine model of surgical biliary obstruction that has not previously been reported. We have achieved a high survival rate to 1 week after BDL with histologic and biochemical findings of obstructive cholestasis. As the immune response differs by age, development of such a neonatal-specific murine model of cholestasis is critical, particularly as etiologies of neonatal cholestasis (e.g. biliary atresia) remain the leading cause for liver transplantation in children. Furthermore, specific etiologies of neonatal cholestasis have distinct differences in disease pathogenesis necessitating multiple models for characterization of the etiology-specific immune response. Our model significantly contributes to the field and enables comparison to the well-established RRV-induced murine model of BA to help identify common features to obstructive cholestasis as well as etiology-specific differences in disease mechanism.

The murine model of bile duct ligation is a well-established model in adult mice resulting in cholestasis, inflammation, parenchymal necrosis, and fibrosis^13^. This model has > 95% survival rate and is highly reproducible, however, a comparable model for neonatal mice has not previously been established^13^. It is particularly important to establish a neonatal-specific disease model due to qualitative and quantitative differences in cell of both the innate and adaptive immune system of neonates compared to older children and adults. Age-specific changes include differences in complement activation, neutrophil migration, inflammatory cytokine production, and lymphocyte differentiation^39–41^. In addition, macrophages in neonates differ by phenotype, function, and response to injury or infection when compared to macrophages in adults^42–44^. For example, while murine neonatal peritoneal macrophages demonstrated increased proinflammatory cytokine secretion after LPS stimulation, they were less able to stimulate T cell proliferation than murine adult peritoneal macrophages^44^. Other murine models of disease, such as those of respiratory disorders, have also demonstrated age-specific differences in illness severity and immune responses, further supporting the importance of disease models that are specific to neonates^45–47^. Our neonatal model of BDL overcomes this gap in knowledge and will help determine how the neonate’s immune response to obstructive cholestasis may differ from adult mice in future studies.

We have effectively used our neonatal model of BDL to identify mechanistic differences from the RRV-induced murine model of BA. In the murine model of BA, mice develop obstructive jaundice at the end of the first week of life which progresses to severe disease by DOL 12–14^17^. To mimic the 1-week duration of progressive biliary injury, we have used a 7-day duration of neonatal BDL for comparison in the present study. Comparison between models demonstrated that despite similar parenchymal injury by Ishak scoring, ALT was significantly higher in neonatal BDL suggesting there may be more subtle differences in the degree or type of liver injury not evident by histology. As macrophages are critical hepatic immune cells present in both parenchymal and portal regions, we evaluated if there were differences in macrophage number between experimental models that would support differences in disease mechanism. Initial analysis by flow cytometry showed lower numbers of Ly6c+ MHCII− macrophages in neonatal BDL versus murine BA suggesting reduced recruitment of Ly6c+ bone-marrow derived monocytes in neonatal BDL. Interestingly, however, the proportion of MHCII+ macrophages did not differ between models, which was unexpected given the established role of antigen presentation in murine BA. These findings demonstrate the need for a more granular analysis of how the immune pathways may differ by experimental model.

Through scRNA-seq we further defined the transcriptional and functional heterogeneity of macrophages across murine models beyond our flow cytometry analysis. We chose to establish this model in BALB/c mice as opposed to another strain to align with the current murine model of BA and reduce inherent immune differences between strains. Both models demonstrated a depletion of tissue resident macrophages as shown through reduction in *Clec4f* across all cholestatic macrophage subsets, but there were also distinct differences that suggest disease-specific adaptation to environmental cues. In line with the known pathophysiology of BA, macrophages in murine BA showed enrichment for processes of antigen presentation, cell killing, and regulation of the immune effector response. In contrast, macrophage clusters in neonatal BDL demonstrated enrichment for processes of cell migration, endocytosis, and biologic processes. As macrophages can be both mal-adaptive or pro-restorative, it is critical to understand both the etiology-specific and subset-specific macrophage functions to uncover disease-specific immune modulatory therapeutic targets. For example, pathogenic macrophage subsets with high antigen-presentation capability in BA may regulate the cytotoxic T cell immune response early in BA disease pathogenesis, whereas macrophage subsets in neonatal BDL may play a critical role in greater immune cell recruitment. Our findings significantly add to prior work that has demonstrated increased macrophage number and activity in murine and human BA but has not defined how their transcriptional heterogeneity differs by etiology as we accomplish in the present study^9–11, 48–51^. Future research, including more mechanistic studies, will determine the precise identity of the pathogenic subsets in each murine model and explore how differences in bile acid composition may impact each disease model.

To enable translation of future studies in murine models to human disease, we also compared our murine cholestatic macrophage subsets to findings in pediatric cholestatic liver disease. We previously identified three distinct human macrophage subsets in children with cholestasis: monocyte-like macrophages, lipid-associated macrophages, and adaptive macrophages^21, 31^. Comparison to murine cholestatic macrophages demonstrated overall reduced expression of immune regulatory genes similar to pediatric cholestatic macrophages. Further subset specific comparisons showed similar gene expression profiles between human monocyte-like macrophages and murine macrophage cluster 0 (*S100* genes and *Vcan*), as well as between human lipid-associated macrophages and murine cluster 1 (*ApoE*, *Lgmn*, *Trem2*)^21, 31^. Through use of murine models, future work will be able to confirm which of these subsets may be bone-marrow monocyte-derived macrophages versus tissue-resident macrophages to identify subset-specific therapeutic targets.

Although the survival rate was high in our study, limitations include the technical challenges associated with performing intricate surgery on small newborn mice. While immune differences and severity of disease may vary by age within the neonatal period, we chose to perform the surgeries at DOL 10 to optimize size and surgical success^52^. We recognize this age is at the end of the neonatal period in mice and aim to overcome technical challenges in the future to apply the model to even younger mice. Additional challenges due to the small size of newborn mice included difficulty in obtaining sufficient blood for flow cytometry which rendered us unable to reliably correlate data between peripheral monocytes and tissue macrophages. We used the same methods for tissue digestion in each model, however, there remains the possibility that tissue preparation may have differentially influenced gene expression in the experimental models. We ran scRNA-seq on pooled samples in each experimental condition. While this technique enables comparison of overall differences between models, we are unable to determine variability between individual replicates. In addition, we merged mouse cells between disease models, which allowed us to determine common cholestatic macrophage subsets and limit the impact of different pipelines over the duration of the study. However, this technique may have detracted from the ability to discern model-specific differences. We also recognize inherent limitations in translating findings from murine models to human disease but anticipate that transcriptional comparisons at the single-cell level will improve this capability. Lastly, our current scRNA-seq focused on hepatic macrophage phenotype in cholestasis. Future work will need to characterize the complete immune cell landscape to fully understand differences in cell–cell interactions between models.

We have developed a successful neonatal murine model of bile duct ligation with low mortality that reproduces features of obstructive cholestasis and differs from the immune-driven murine model of BA. Through use of scRNA-seq, we show that murine cholestatic macrophages have reduced expression of immunoregulatory genes similar to hepatic macrophages in pediatric cholestasis. Furthermore, we identified etiology-specific macrophage functions through comparison of neonatal BDL and murine BA. Future research will include validation studies of our macrophage subsets by histology, time point data, the inclusion of alternate neonatal models such as reversible models of BDL and the RRV reassortant-induced murine model of liver fibrosis, and comparison of our model to adult models to provide a deeper understanding of age-specific differences in the neonatal immune response to obstructive cholestasis^8, 53^. Ultimately by translating findings from our novel neonatal model of BDL to children with cholestasis, we hope to identify disease-specific and macrophage subset-specific immunomodulatory therapies to improve patient outcomes.

## Supplementary Information

Supplementary Figure 1.

Supplementary Figure 2.

Supplementary Figure 3.

Supplementary Figure 4.

Supplementary Figure 5.

Supplementary Figure 6.

Supplementary Table 1.

Supplementary Table 2.

Supplementary Table 3.

## Abbreviations

BA: Biliary atresia
MΦ: Macrophage
BDL: Bile duct ligation
DOL: Day of life
RRV: Rhesus rotavirus
scRNA-seq: Single-cell RNA-sequencing
ALT: Alanine aminotransferase
DAMP: Disease associated molecular pattern
ALP: Alkaline phosphatase
TB: Total bilirubin
H&E: Hematoxylin and eosin
FMO: Fluorescence minus one
UMAP: Uniform Manifold Approximation and Projection
GO: Gene ontology
SEM: Standard error of the mean
