# Supplementary material for: Single-cell sequencing of a novel model of neonatal bile duct ligation in mice identifies macrophage heterogeneity in obstructive cholestasis
Source: Sci Rep. 2023 Aug 29;13:14104. doi: 10.1038/s41598-023-41207-0 (PMC10465511; doi:10.1038/s41598-023-41207-0)
Supplement: Supplementary file 2 — Supplementary Figure 2. [file 41598_2023_41207_MOESM2_ESM.pdf]

A.

	Number of Cells	Median UMI Counts/Cell	Median Genes/Cell	Reads Mapped to Genome	% Reads in Cells
**Control**	8,172	4,754	1,850	87.8%	90.8%
**BDL**	5,509	13,262	3,461	95.9%	87.4%
**RRV**	5,185	8,138	2,365	87.0%	84.6%

B.

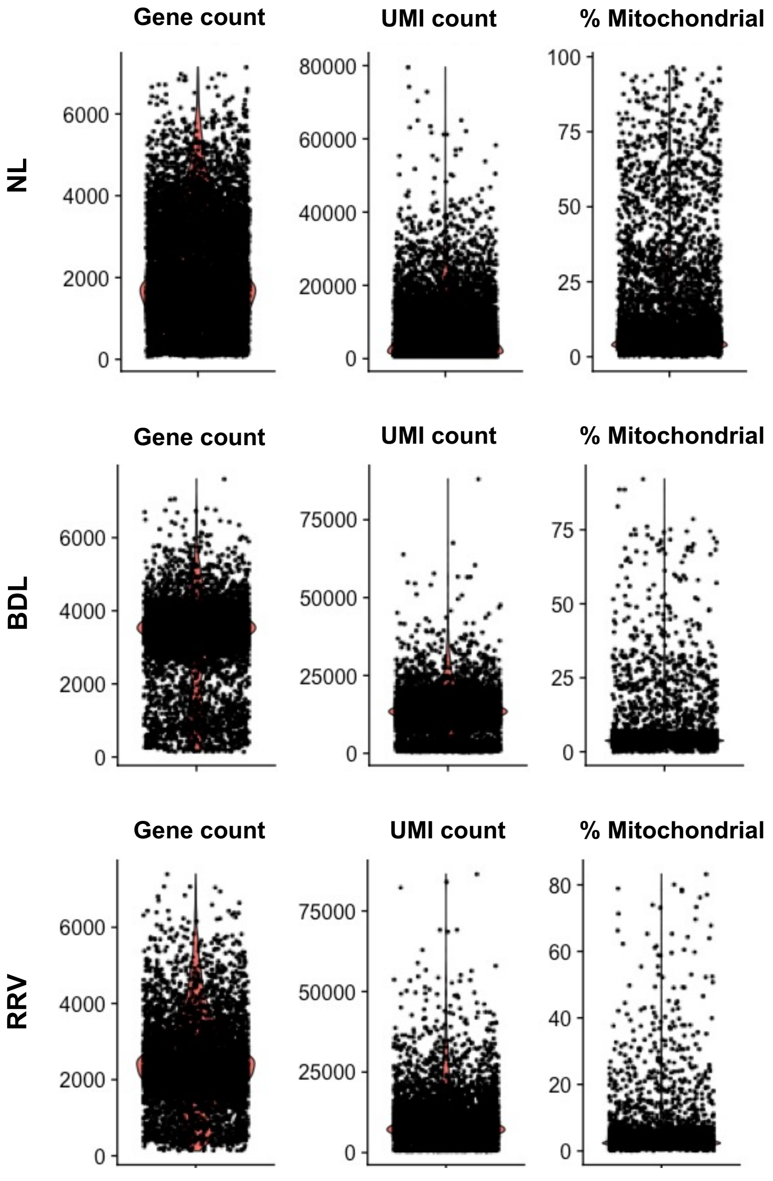

Supplemental Figure 2. A. scRNA-seq results from each murine model. **B.** Gene counts, UMI counts, and % mitochondrial genes for each murine model before filtering.
