# Supplementary material for: Single-cell sequencing of a novel model of neonatal bile duct ligation in mice identifies macrophage heterogeneity in obstructive cholestasis
Source: Sci Rep. 2023 Aug 29;13:14104. doi: 10.1038/s41598-023-41207-0 (PMC10465511; doi:10.1038/s41598-023-41207-0)
Supplement: Supplementary file 4 — Supplementary Figure 4. [file 41598_2023_41207_MOESM4_ESM.pdf]

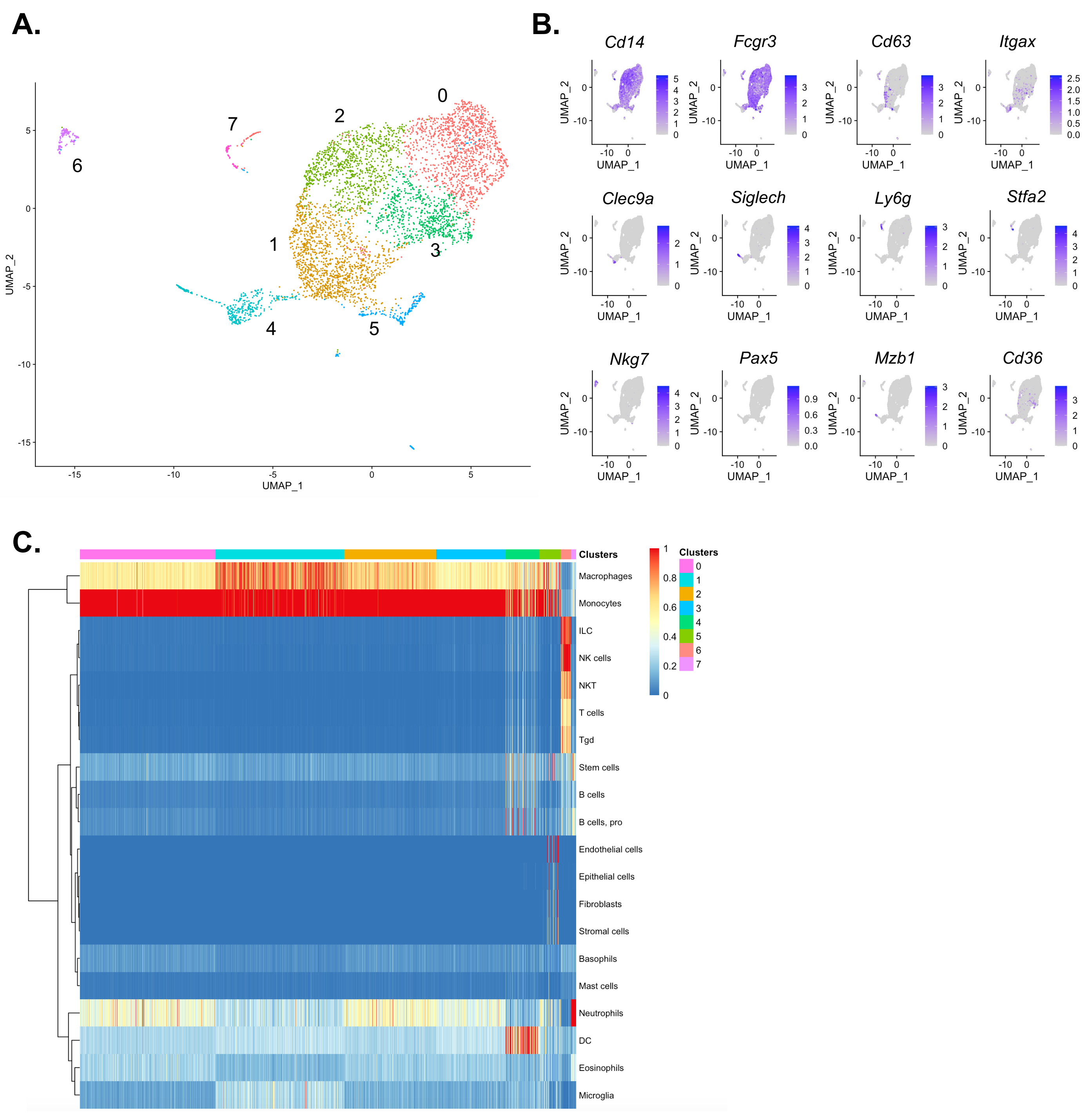

Supplemental Figure 4. A-B. Clustering of neonatal BDL murine liver cells and visualization of canonical cell surface markers across clusters. **C.** Single-R analysis confirmed cluster assignments.
