# Supplementary material for: Single-cell sequencing of a novel model of neonatal bile duct ligation in mice identifies macrophage heterogeneity in obstructive cholestasis
Source: Sci Rep. 2023 Aug 29;13:14104. doi: 10.1038/s41598-023-41207-0 (PMC10465511; doi:10.1038/s41598-023-41207-0)
Supplement: Supplementary file 6 — Supplementary Figure 6. [file 41598_2023_41207_MOESM6_ESM.pptx]

## Slide 1
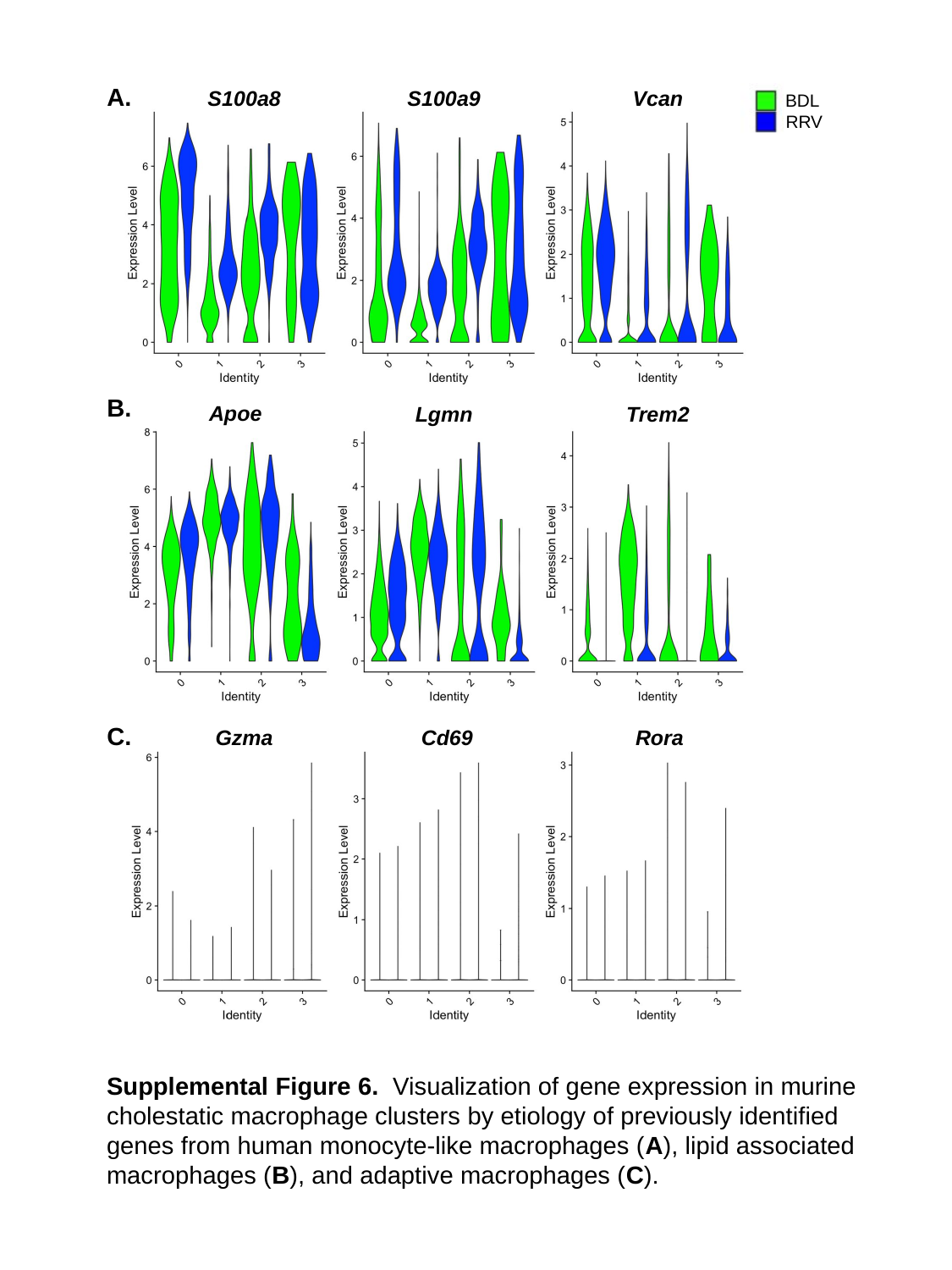

A.
BDL
RRV
Vcan
S100a8
S100a9
B.
Apoe
Lgmn
Trem2
C.
Gzma
Cd69
Rora
Supplemental Figure 6. Visualization of gene expression in murine cholestatic macrophage clusters by etiology of previously identified genes from human monocyte-like macrophages (A), lipid associated macrophages (B), and adaptive macrophages (C).
