# Supplementary material for: Single-cell sequencing of a novel model of neonatal bile duct ligation in mice identifies macrophage heterogeneity in obstructive cholestasis
Source: Sci Rep. 2023 Aug 29;13:14104. doi: 10.1038/s41598-023-41207-0 (PMC10465511; doi:10.1038/s41598-023-41207-0)
Supplement: Supplementary file 7 — Supplementary Table 1. [file 41598_2023_41207_MOESM7_ESM.docx]

Supplemental Table 1. Mouse Flow Antibodies

| **Antibody** | **Manufacturer** | **Fluorochrome** | **Host** | **Clone** | **Concentration per 5 million cells** |
| --- | --- | --- | --- | --- | --- |
| Anti-CD11b | BD Biosciences | BV421 | Rat | M1/70 | 0.009 |
| Anti-CD45 | BD Biosciences | FITC | Rat | 30-F11 | 0.001 |
| Anti-MHCII | Biolegend | PerCP/Cy5.5 | Rat | M5/114.15.2 | 0.01 |
| Anti-CD64 | BD Biosciences | PE | Mouse | X54-5/7.1 | 0.3 |
| Anti-Siglec-F | BD Biosciences | PE-CF594 | Rat | E50-2440 | 0.7 |
| Anti-Ly6g | BD Biosciences | PE-CF594 | Rat | 1A8 | 0.03 |
| Anti-F4/80 | Biolegend | PE/Cy7 | Rat | BM8 | 0.7 |
| Anti-CD11c | Biolegend | APC | Armenian Hamster | N418 | 0.06 |
| Anti-Ly6c | BD Biosciences | APC-Cy7 | Rat | AL-21 | 0.05 |
| Anti-CD3 | BD Biosciences | AF 700 | Hamster | 500A2 | 0.7 |
| Anti-CD19 | BD Biosciences | AF 700 | Rat | 1D3 | 1.0 |
